# Supplementary material for: Patterns of Intron Gain and Loss in Fungi
Source: PLoS Biol. 2004 Nov 30;2(12):e422. doi: 10.1371/journal.pbio.0020422 (PMC532390; doi:10.1371/journal.pbio.0020422)
Supplement: Table S1 — Also available at http://genes.mit.edu/NielsenEtAl/. (4.3 MB ZIP). [file pbio.0020422.st001.zip › NielsenEtAl/html/999.html]

AN6711.1.NCU05254.1.MG01828.1.FG04261.1


```
 CLUSTAL W (1.82) Multiple Sequence Alignments - Introns Inserted


Sequence 1: NCU05254.1	485 aa
Sequence 2: MG01828.1	503 aa
Sequence 3: FG04261.1	502 aa
Sequence 4: AN6711.1	462 aa
Alignment Length: 527 aa
Number Identitical Residues: 210 aa
Alignment Score (without introns) 10063


MG01828.1 	MRNTLIFSGTSCPDLTKRICENLGMAPAEAELTQFSN0GETSVRILTSTREKDVFVVQSG
NCU05254.1	MRGTLIFSGSSCPALTGKVCENLGMTPADAELTQFSN~GETSVRILTSVREKDVFVVQSG
FG04261.1 	MRNTLIFAGNSCPVLTGQICENLGMHPASAELTQFSN0GETSVRILTSVREKDVFVVQSG
AN6711.1  	MRGVQIFSGNSHPLLTETVCERLGTSPAKVELGKFSN~GETNVNVGVSVRNQDVYIIQSG
          	**.. **:*.* * **  :**.**  **..** :*** ***.*.: .*.*::**:::***

MG01828.1 	SPKINDTIMELLIMISACKGGSASKITA1VLPYFPYNRQSKKKSHRGAITARMLANLLHV
NCU05254.1	SPKINDTIMELLIMISACKGGSASKITA1VLPYFPYSRQSKKKSHRGAITARMLANLLGV
FG04261.1 	SPSINDSIMELLIMISACKGGSANKVTA1VLPYFPYSRQSKKKSHRGAITARMLANLLGV
AN6711.1  	SEKINDSVMELLIMISACKGGSAKSIT-~------------------------------V
          	* .***::***************..:*                                *

MG01828.1 	~AGVKHVITVDLH~ASQMQGFFQCPVDNLHAEPLIARWIRLNVSNWREAVVVSKNAGGTK
NCU05254.1	~AGVKHIITVDLH~ASQMQGFFKCPVDNLHAEPLIARWIRQNVPDWKEAVVVSKNAGGTK
FG04261.1 	~AGVKHIITVDLH~ASQMQGFFKCPVDNLHAEPILAKWIRHNVSNWREAVVVSKNAGGTK
AN6711.1  	1AGVDHVITLDLH0ASQMQGFFSKPVDNLFAEPFIARWIRNNVPGWKEAVVVSKNAGGTK
          	 ***.*:**:*** ********. *****.***::*:*** **..*:*************

MG01828.1 	RVTSLADALKLNFGMVTTDKKRGGGNMTASMILEQLDRP---DPVAVPTKDGGVATTP--
NCU05254.1	RVTSLADALKLNFGMVTTDKKRGNGNMTGSMLLNPIDAVL--DPIGEQVNEAVEHEEP--
FG04261.1 	RVTSLADALKLNFGIVTTDRKR-VSNMTASMIMRHFDHNVDRQPTPLESNRPIPYRGPPQ
AN6711.1  	RVTSLADTLKLNFGIVTTDRRRQKPGPT------LADSTVFFEALDQETTPLPKEQDPFI
          	*******:******:****::*   . *        *     :.     .       *  

MG01828.1 	TAEAPFPNLDGTSEATSSKTRVVADSRGSPKRIPVRTNGRSRVTSHPIQTETVAEEKEQA
NCU05254.1	SVSNITQPIAGT---TSPARRPNGSSMHSAKSLP------SRLRSSTSALDGVDEE----
FG04261.1 	AERAVETDVTPKRETPPPRTRIVTNSQGSPTRVDS-----SARVPDPNMTEPVDSNATPP
AN6711.1  	ARTHIEN----------PDALQSRESQADLNELDS-------LRPETPSGARRPSE----
          	:                .      .*  . . :           . .       .:    

MG01828.1 	EGDVVDNGEDADDENDGRPDEVTHARLFQGHIVPDDFP-------------SPSTSAADA
NCU05254.1	-------GEDGVEYNDQRAHEVTQARLVQGHIVPDDFE-------------SPAVSAATS
FG04261.1 	AGSPDDSVNGETDYDDHKASEVTQGRLVQGRIVEDDYP-------------SPDRSVVDG
AN6711.1  	-------LEAVNEYTDVRVRDVITGRLVQGHLVDDDYPPSGPTSMAVSGDATPGQLPSQE
          	        :   :  * :  :*  .**.**::* **: .:..:: : :..::*       

MG01828.1 	SLPDDDPMMMSHASSFFQPDNH-ALGGSGDAA-SSDEEEDKLK-DPRIEHMITLVGNVRN
NCU05254.1	-VAGDDPMTMSHASSFFAPQHHAALGGSGDAAESSDEEEEILK-DPKVEHMVTLVGDVKN
FG04261.1 	-SVDDDPMTMSHASSFFVPEPQ-SLGGSGDAAASSDEEDNAFE-EPGAEHLITLVGNVKN
AN6711.1  	SEAIPDSMVDSVVSTTSSMPGDHALGGSFDAADSSDEEEGSTKRNVEQEKTITLVGDVRD
          	:    *.*  * .*:      . :**** *** *****:   : :   *: :****:*::

MG01828.1 	RTVFIVDDMIDKPGSWIAAAEAVVKRGGATTVYCMATHGVFGGDCLEQLEACDCIHRIVV
NCU05254.1	RTVFIVDDMIDKPGSWIAAAETVVKRGLAKKVYCIATHAVFGADCLEQLQDCDCIDHILV
FG04261.1 	RTVFIVDDMIDKAGSWIAAAETVVKKGGAKRVYCMATHGVFGGDSLEQLQACECIDQIVV
AN6711.1  	RTVFLVDDMIDKSGSWIAAAETVVKRGGAKKVYCIATHGLFGDSSLEQLEACESINYIVV
          	****:*******.********:***:* *. ***:***.:** ..****: *:.*. *:*

MG01828.1 	TDSYPIRPEKARSASKLVILDLSHLLAEAIRRNHNGEA~---ISPLFQ~HIVD
NCU05254.1	TNSYPIPEEKARNASKLTVLDLSYLLAEAIRRNHYGES~---ISPLFQ~HIME
FG04261.1 	TNSFPIDKDRARSISKLVVLDLSFLLAEAIRRNHYGEA~---LSPLFQ~HYGD
AN6711.1  	TNTFPISPQVARKSRKLIIIDISSLLSESIRRHHYGES2DWAVCNLHD1YEGR
          	*:::**  : **.  ** ::*:* **:*:***:* **: . ::. *.: :
```
